# Modeling Exact Frequency-Energy Distribution for Quakes by a Probabilistic Cellular Automaton

**DOI:** 10.3390/e25050819

**Published:** 2023-05-19

**Authors:** Mariusz Białecki, Mateusz Gałka, Arpan Bagchi, Jacek Gulgowski

**Affiliations:** 1 Institute of Geophysics Polish Academy of Sciences, 01-452 Warsaw, Poland; 2 The Faculty of Mathematics, Physics and Informatics, University of Gdańsk, 80-308 Gdańsk, Polandjacek.gulgowski@ug.edu.pl (J.G.)

**Keywords:** modeling, earthquake statistics, magnitude-frequency distribution, modeling Gutenberg-Richter law, probabilistic cellular automata, solvable models, stochastic processes, toy model of earthquakes

## Abstract

We develop the notion of Random Domino Automaton, a simple probabilistic cellular automaton model for earthquake statistics, in order to provide a mechanistic basis for the interrelation of Gutenberg–Richter law and Omori law with the waiting time distribution for earthquakes. In this work, we provide a general algebraic solution to the inverse problem for the model and apply the proposed procedure to seismic data recorded in the Legnica-Głogów Copper District in Poland, which demonstrate the adequacy of the method. The solution of the inverse problem enables adjustment of the model to localization-dependent seismic properties manifested by deviations from Gutenberg–Richter law.

## 1. Introduction

Earthquakes are among the most devastating natural phenomena, but their studies are far from being completed, mainly because the mechanisms of earthquakes are still not fully understood [1]. They are extremely complex, and investigation of them involves many different approaches (see, for example, [2]). There is also additional complexity coming from sensitivity on local geological settings, which are always—to some extent—unknown. In spite of dependence on local tectonics, there are known universal empirical laws for earthquakes—Gutenberg–Richter (G-R) law [3], and Omori law [4]. Earthquakes, being complex systems, exhibit scaling behavior [5], in particular the probability density function of interevent times can be rescaled into a single function [6,7,8]. This property was further investigated in the context of the epidemic-type aftershock sequence (ETAS) models [9,10]. Other universal behavior of earthquakes was investigated using a concept of natural time—see [11,12] and references therein.

This article is a contribution toward constructing a mechanistic model in the form of probabilistic cellular automata (PCAs) for generating interevent time distribution based on a locally deviated size-frequency distribution (see also [13]). In this paper, we focus on accurate modeling of the frequency-energy distribution, while time-related issues will be presented in the next article.

PCAs [14] are simple computational models, yet are capable of simulating complex phenomena. In particular, cellular automata proved to be useful modeling tools in seismology [15]. The action of CA and PCA is determined by its local transformation rules, which—in the case of natural phenomena—reflect the physical dependencies crucial for the phenomenon under investigation. Their relatively simple structure allows for insight into expected time series and general properties of earthquakes.

Here, we consider abstract representation of earthquake statistics by Random Domino Automaton (RDA) [16] with a local transformation rule based on a dissipative process of slow energy accumulation and abrupt releases understood as quakes. Contrary to the majority of PCA models, which are investigated mostly by extensive simulations (see, for example, [17] and also references therein), RDA possesses a unique mathematical structure, which allows for analytical derivation of several of its properties and constraints in the form of equations for stationary state.

The plan of this paper is as follows. In Section 2, we introduce the notion of Random Domino Automaton. Section 3 contains the derivation of equations describing the stationary state of the automaton. Some detailed calculations of the derivation are presented in Appendix A. Next, in Section 4, we formulate and solve the inverse problem for Random Domino Automaton. Section 5 contains application of the developed framework to exemplary open access data of episode LGCD [18] from LUMINEOS network located in the Legnica-Glogow Copper District, Poland (for details see [19,20]). Finally, we end with a discussion in Section 6.

## 2. The Model

### 2.1. The Random Domino Automaton

The RDA model [16] is a completely discrete dynamical system, i.e., both independent and dependent variables are discrete. Space consists of *N* cells along a line or circle, if periodic boundary conditions are assumed. This means that a cell has exactly two neighboring cells. The size of the automaton is *N*, where we assume *N* can be arbitrarily big.

Each cell may be empty or occupied by a single ball, which represents the absence or presence of energy, respectively. If a number *i* of consecutive cells are occupied, they belong to a cluster of size *i*. Clusters are separated by empty clusters—consecutive empty cells. The size of an empty cluster is equal to the number of cells contained in it. Empty cells may have zero, one or both neighboring cells occupied, and hence we distinguish three kinds of them. We will refer to these three kinds of cells as creation, enlarging and merging cells, respectively. The names originate from the dynamics of the automaton: what is the influence of the change in the status of the cell (from empty to occupied) on the total number of clusters?

### 2.2. Evolution Rules

Discrete time dynamics is defined as follows. In each time step one cell is chosen. We assume that every cell has the same chance of being selected.

- If the chosen cell is empty, then it becomes occupied with some fixed probability, depending of the type of the cell. The value of probability is: $c_{0}$ for a creation cell, $c_{1}$ for an enlarging cell, and $c_{2}$ for a merging cell. The state of the chosen cell remains unchanged (i.e., empty) with probability $\left(1-c_{0}\right)$, or $\left(1-c_{1}\right)$, or $\left(1-c_{2}\right)$, respectively.
- If the chosen cell is occupied, and it belongs to the cluster of size *i*, the whole cluster is removed (i.e., each cell in the cluster is emptied) with probability $\mu \left(i\right)=\mu_{i}$ depending on the size *i* of the cluster. The state of the chosen cell stays the same (i.e., occupied) with probability $\left(1-\mu_{i}\right)$.

The removed cluster forms an avalanche of size equal to the size of the removed cluster.

For μ=constant the RDA model is equivalent to the Drossel–Schwabl forest fire model [21].

## 3. Equations

### 3.1. Variables

The number of clusters of size *i* in the system is denoted by $n_{i}$, and the number of empty clusters of size *i* by $n_{i}^{0}$. Both $n_{i}$s and $n_{i}^{0}$s depend on time; however, we will refer to these variables as describing a stationary state, thus constant [22]. Note that these constant values are average values over time, and hence they are non-negative real numbers, not necessarily integers.

The density of the system, a ratio of the number of occupied cells to the size of the system, is thus given by

(1) $$\rho =\frac{1}{N}\sum_{i\geq 1}in_{i}.$$

Energy of a cluster of size *i* is equal to *i* statutory units, and hence ρN refers to the total energy contained in the system. The total number of clusters is

(2) $$n=\sum_{i\geq 1}n_{i}.$$

The total number of empty clusters $n^{0}$ is defined in the same way.

We assume periodic boundary conditions, i.e., assume that the last cell is adjacent to the first one, and also, that there are at least one cluster and at least one empty cluster present in the system. These assumptions make *n* and $n^{0}$ equal to each other, because each cluster is followed by an empty cluster. Without these assumptions, the actual values of *n* and $n^{0}$ can either be equal to each other or may differ by one. For the large size of the system *N*, and large values of *n* and $n^{0}$, such a difference is negligible.

There are three kinds of empty cells, distinguished according to the number of occupied neighbors. An empty cell may be a neighbor for 0, 1 or 2 occupied cells. The total number of such empty cells in the system is denoted by $x_{0}$, $x_{1}$ and $x_{2}$, for these three kinds, respectively. Therefore, it follows

(3) $$\sum_{i\geq 1}in_{i}^{0}=x_{0}+x_{1}+x_{2}=\left(1-\rho \right)N.$$

An empty cell can change its state to occupied, according to specific evolution rules defined below. In such a case, there are three different situations, depending on the number of its occupied neighbors. Occupation of an empty cell with no occupied neighbor results in the creation of a new cluster (of size 1). Other results are: enlarging of an existing adjacent cluster and merging of two adjacent clusters. Hence, empty cells are named: creating, enlarging and merging, respectively.

The following expressions for $x_{i}$, i=0,1,2 follow directly from the definitions

(4) $$\begin{array}{ccc} x_{0} & = & \sum_{i\geq 3}\left(i-2\right)n_{i}^{0}=\left(1-\rho \right)N-2n+n_{1}^{0}, \end{array}$$

(5) $$\begin{array}{ccc} x_{1} & = & 2\sum_{i\geq 2}n_{i}^{0}=2\left(n-n_{1}^{0}\right), \end{array}$$

(6) $$\begin{array}{ccc} x_{2} & = & n_{1}^{0}. \end{array}$$

The first one comes from counting empty cells from interiors (inner cells, without those on ends) of empty clusters and the second from counting the remaining end cells.

We point out the identity

(7) $$n=\frac{1}{2}\left(x_{1}+2x_{2}\right),$$

which reflects the fact that each cluster has two empty cells as neighbors. These cells are of enlarging or merging type, and each merging cell is a neighbor for two clusters. Obviously, the constraint expressed by the Equation (7) follows also from Equations (5) and (6).

### 3.2. Equations

The RDA is a Markov chain, and its space of states is irreducible, aperiodic and recurrent [22]. Thus, statistically stationary state is well defined, and it is possible to derive respective balance equations by using the “flow in = flow out” principle, and counting respective probabilities.

Below, we present the balance equations for ρ, *n*, $x_{i}$ and $n_{i}$ for the statistically stationary state of the automaton in mean field approximation for the special choice $c_{0}=c_{1}=c_{2}=c$, while details of the derivation in the general case (arbitrary $c_{0}$, $c_{1}$ and $c_{2}$) are presented in Appendix A.

The balance equation for density ρ reflects an equilibrium condition between all processes—creating, enlarging, merging and triggering avalanches, and hence it is

(8) $$c\left(x_{0}+x_{1}+x_{2}\right)=\sum_{i\geq 1}\mu_{i}n_{i}i^{2}.$$

The balance equation for the number of clusters *n* is

(9) $$c\left(x_{0}-x_{2}\right)=\sum_{i\geq 1}\mu_{i}n_{i}i.$$

All processes, except for enlarging, make a change to the number of clusters.

These two equations are exact, contrary to the following equations, whose derivation uses the mean field approximation (see Appendix A). It follows that the balance equation for creating cells $x_{0}$, enlarging cells $x_{1}$ and merging cells $x_{2}$ are of the form

(10) $$\begin{array}{ccc} 3cx_{0} & = & \sum_{i\geq 1}\mu_{i}n_{i}i^{2}+\frac{x_{1}}{n}\sum_{i\geq 1}\mu_{i}n_{i}i, \end{array}$$

(11) $$\begin{array}{ccc} 2c(x_{0}-x_{1}) & = & \frac{x_{1}-2x_{2}}{n}\sum_{i\geq 1}\mu_{i}n_{i}i, \end{array}$$

(12) $$\begin{array}{ccc} c(x_{1}-x_{2}) & = & \frac{2x_{2}}{n}\sum_{i\geq 1}\mu_{i}n_{i}i. \end{array}$$

**Remark 1.**  *Not all of the equations derived above are independent. Because of the relation (3), a combination of Equations (10)–(12) gives the Equation (8) for the density ρ. The relation (7), implies that a combination of Equations (11) and (12) must be consistent with the Equation (9) for the total number of clusters n.*

Finally, the balance equations for $n_{i}$’s are as follows:

(13) $$\begin{array}{cc} n_{1} & =\frac{1}{\mu_{1}+2}\left(cx_{0}\right), \end{array}$$

(14) $$\begin{array}{cc} n_{2} & =\frac{1}{2\mu_{2}+2}\left(cx_{1}\frac{n_{1}}{n}\right), \end{array}$$

(15) $$\begin{array}{cc} n_{i} & =\frac{1}{i\mu_{i}+2}\left(cx_{1}\frac{n_{i-1}}{n}+cx_{2}\sum_{k=1}^{i-2}\frac{n_{k}}{n}\frac{n_{i-k-1}}{n}\right)\;\mathrm{for}\;i\geq 3. \end{array}$$

**Remark 2.**  *Equations (13)–(15) sum up to balance Equation (8) for ρ.*

## 4. Inverse Problem

### 4.1. Formulation

The inverse problem for RDA [23] is to find the rebound parameters (probabilities $\mu_{i}$ and $c_{i}$) that result in the given stationary distribution of avalanches $w_{i}$. Here, $w_{i}$ is a frequency of appearance of avalanches of a given size *i*.

The probability of an avalanche of size *i* is proportional to the number of cells contained in clusters of size *i* (i.e., $i\cdot n_{i}$) times respective rebound parameter $\mu_{i}$, hence

(16) $$w_{i}∼\mu_{i}n_{i}i,$$

and we assumed a normalization $\sum_{i}w_{i}=1$.

A key observation for solving the inverse problem is that it is possible to express parameters of $\mu_{i}$ as functions of $n_{i}$ and $x_{i}$, using Equations (13)–(15). Moreover, the set of Equations (26)–(28), which are shown below, allows us to calculate $w_{i}$ from $n_{i}$ sequentially. In order to achieve this goal, we need one more step, namely defining “unit-less” variables.

Consideration of the stationary state makes the “speed” of time flow irrelevant, and thus the stationary state of RDA is influenced by ratios of probabilities rather then values of rebound parameters itself. Similarly, considering relative frequencies of avalanches, we may remove the dependence of numbers of avalanches and clusters in favor of ratios of respective variables in a way analogous to definition “intensive” (as opposite to “extensive”) variables in thermodynamics and statistical physics. Thus, we define

(17) $${\hat{n}}_{i}:=\frac{n_{i}}{n},\;{\hat{x}}_{i}:=\frac{x_{i}}{n},\;\mathrm{and}\;{\hat{\mu }}_{i}:=\frac{\mu_{i}}{c}.$$

Note $0\leq {\hat{x}}_{1}\leq 2$, $0\leq {\hat{x}}_{2}\leq 1$, and ${\hat{x}}_{0}$, $\hat{\mu }$ are non-negative.

Using these “unit-less” variables, we define

(18) $$I:=\sum_{i\geq 1}{\hat{\mu }}_{i}{\hat{n}}_{i}i,\;\mathrm{and}\;J:=\sum_{i\geq 1}{\hat{\mu }}_{i}{\hat{n}}_{i}i^{2},$$

where we assume both sums are convergent. Notice that the average size of avalanche $\eta :=<i_{w}>$ is

(19) $$\eta :=<i_{w}>=\frac{\sum_{i}\mu_{i}n_{i}i^{2}}{\sum_{i}\mu_{i}n_{i}i}=\frac{\sum_{i}{\hat{\mu }}_{i}{\hat{n}}_{i}i^{2}}{\sum_{i}{\hat{\mu }}_{i}{\hat{n}}_{i}i}=\frac{J}{I}.$$

Obviously η≥1.

In view of Remark 1 and the relation (7)

(20) $${\hat{x}}_{1}=2\left(1-{\hat{x}}_{2}\right),$$

which allows us to eliminate the variable ${\hat{x}}_{1}$, the set of balance equations is reduced to three independent equations. The balance Equations (8)–(12) (for the density ρ, for the total number of clusters *n*, and for the number of merging cells $x_{2}$) expressed in normalized variables are of the form

$$\begin{array}{ccc} 2+{\hat{x}}_{0}-{\hat{x}}_{2} & = & J,\\ {\hat{x}}_{0}-{\hat{x}}_{2} & = & I,\\ 2-3{\hat{x}}_{2} & = & 2{\hat{x}}_{2}I. \end{array}$$

The set can be solved explicitly

(21) $$\begin{array}{ccc} {\hat{x}}_{0} & = & \frac{2}{\eta -1}+\frac{2}{3}\left(1-\frac{4}{3\eta +1}\right), \end{array}$$

(22) $$\begin{array}{ccc} {\hat{x}}_{2} & = & \frac{2}{3}\left(1-\frac{4}{3\eta +1}\right), \end{array}$$

(23) $$\begin{array}{ccc} {\hat{x}}_{1} & = & \frac{2}{3}\left(1+\frac{8}{3\eta +1}\right). \end{array}$$

and

(24) $$I=\frac{2}{\eta -1},\;\;J=\frac{2\eta }{\eta -1}.$$

Note that the limit η⟶∞ gives ${\hat{x}}_{i}=2/3$ for i=1,2,3. Note also that the average size of empty clusters is

(25) $$<i_{n}^{0}>=2+\frac{2}{\eta -1}.$$

We can also see that, from the equations of the distribution of clusters (13)–(15), by removing denominators and rearranging, one can obtain

(26) $$\begin{array}{cc} {\hat{n}}_{1} & =\frac{1}{2}\left({\hat{x}}_{0}-w_{1}I\right), \end{array}$$

(27) $$\begin{array}{cc} {\hat{n}}_{2} & =\frac{1}{2}\left({\hat{x}}_{1}{\hat{n}}_{1}-w_{2}I\right), \end{array}$$

(28) $$\begin{array}{cc} {\hat{n}}_{i} & =\frac{1}{2}\left({\hat{x}}_{1}{\hat{n}}_{i-1}+{\hat{x}}_{2}\sum_{k=1}^{i-2}{\hat{n}}_{k}{\hat{n}}_{i-k-1}-w_{i}I\right)\;\mathrm{for}\;i\geq 3. \end{array}$$

Summing up these equations for all *i*, one can obtain the identity 0=0.

### 4.2. The Procedure of the Solution to the Inverse Problem

With equations of the previous subsection, we can formulate the following procedure of solving the inverse problem.

- Take $w_{i}$ from data and normalize $\sum_{i}w_{i}=1$.
- Calculate the average size of avalanche $\eta =<i_{w}>$.
- From Equation (24), calculate *I*.
- From (21)–(23), calculate ${\hat{x}}_{0}$, ${\hat{x}}_{1}$ and ${\hat{x}}_{2}$.
- From Equations (26)–(28), calculate ${\hat{n}}_{i}$.
- Rebound parameters $\mu_{i}$ follow from the formula (16), namely ${\hat{\mu }}_{i}:=\frac{Iw_{i}}{{\hat{n}}_{i}i}$.

## 5. Applications

### 5.1. Exponential Tail

First, we apply the proposed above framework to an avalanche distribution, given by a geometric relation:

(29) $$\begin{array}{ccccc} w_{i}=\left(\frac{1-q}{q}\right)q^{i}, & \; & 0<q<1, & \; & i=1,2,\cdots \end{array}$$

We set q=99/100, which implies the average size of avalanche η=100, and having performed computations using rational values, one can obtain the following *exact* values:

(30) $$I=\frac{2}{99},\;{\hat{x}}_{0}=\frac{20204}{29799},\;{\hat{x}}_{1}=\frac{206}{301},\;{\hat{x}}_{2}=\frac{198}{301}.$$

**Figure 1:**
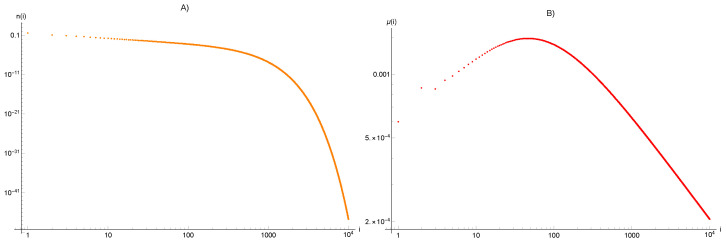
The distribution of clusters ${\hat{n}}_{i}$ (**A**) and the distribution of rebound parameters $\mu_{i}$ (**B**) calculated for avalanche size distribution $w_{i}$ given by geometric series of Equation (29) with q=99/100.

The distribution of clusters ${\hat{n}}_{i}$ is displayed in Figure 1 on the left side. The starting two values equal ${\hat{n}}_{1}$ = 10,201/30,100 and ${\hat{n}}_{2}$ = 104,979,699/906,010,000. The sequence of parameters $\mu_{i}$ obtained accordingly is calculated up to i=10.000 and presented in Figure 1 on the right side.

Starting with obtained values of rebound parameters $\mu_{i}$ and calculating values of $w_{i}$ from respective equations, we reconstruct the values of avalanche distribution. Due to the usage of computations with rational numbers, which contain no approximation errors, the distribution fits to the geometric series (29) with value q=99/100 *exactly*. This property, that geometric extinction can be calculated exactly, makes it useful for serving as a smooth cut-off of any real data, which always are given within some finite range of values.

### 5.2. Seismic Data from LUMINEOS Network

Next-generic-example deals with exemplary data [18] recorded by LUMINEOS Seismic Network in the Legnica-Głogów Copper District in Poland. The open-access data were downloaded from and are available on the EPISODES platform [19] within LGCD episode [20]. The data set contains 6095 items, with 32 different values of magnitude, ranging from M0.9 to M4.0, rounded to the nearest 0.1. The catalogue completeness threshold—i.e., the minimal value of magnitude above which 100% of the earthquakes are detected—is estimated to M1.7. This value is relatively high as for the local seismic network, and it reflects the influence of high noise levels generated by industrial activity nearby. We emphasize that the modeling data below the catalog completeness threshold do not reflect the physical properties of the system. However, we also model these data to show the scope of the model’s capabilities.

The notion of Random Domino Automaton assumes that the sizes of avalanches and clusters correspond to energy, thus we convert magnitudes *M* to energies *E* using the formula [24]

(31) $$E=C\cdot 10^{1.5M},$$

where *C* is a constant, and its value depends on units of energy. Assigning a value of energy to a single occupied cell is arbitrary, so we use this freedom and set C=0.05. This choice gives for the lowest recorded magnitude value M=0.9 the value of E=1.1, for the catalog completeness threshold M=1.7 it gives E=17.7, and $E=5\cdot 10^{4}$ for the highest magnitude value M=4.0. We interpret a magnitude *M* as cumulative value for the interval (M−0.05,M+0.05), so the maximal value of energy is 59,425. On the other side, with the resulting low resolution for the smallest energies—which are also well bellow the catalog completeness threshold—the first two values corresponding to M0.9 and M1.0 we treat as a contributing to a single record.

Next, we take these 31 values as a basis for piece-wise linear interpolation at [1, 59,425], and we treat the obtained spline function as frequency distribution. It is also possible to choose another interpolation, however for our purpose to show possibility of reproducing variability of energy-frequency distribution, this is not of the primary importance. The piece-wise interpolation contains artificially created abrupt changes in the decrease rate, which is rather more difficult to reproduce, then only smooth changes.

Then, we fit inverse power distribution to the spline starting with value *i* = 42, which is equivalent to magnitude M1.95, and even slightly more than the provided catalog completeness threshold M1.7. We use the procedure of [25], to fit a truncated power-law, by logarithmic binning, maximizing the highest-likelihood estimator with data values parametrized along, to fit the distribution at the theoretical centers of weight of 18 intervals with the downhill simplex numerical method (see [25] for details). We obtain the following inverse-power distribution:

(32) $$f\left(i\right)=53122.9\cdot i^{-1.854}.$$

**Figure 2:**
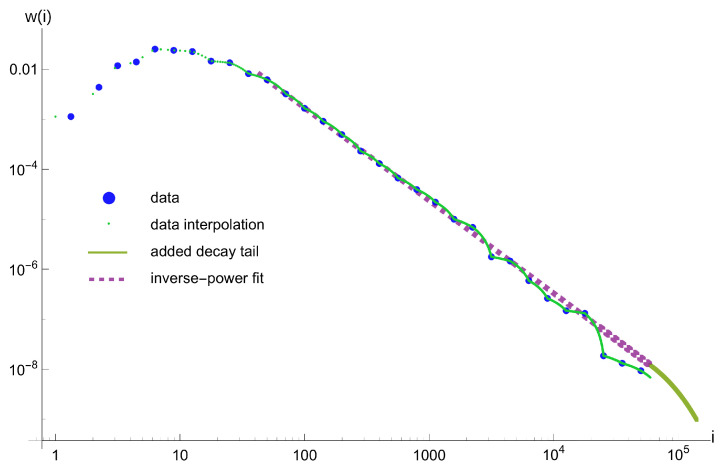
The augmented distribution of avalanches $w_{i}$, up to *i* = 140,000, obtained from the LGCD episode from Legnica-Głogów Copper District in Poland. The recorded data (blue dots) were adjusted to the resolution of RDA model (green dots gradually turning into a line) and the exponential tail (thicker green line) was added using the auxiliary inverse-power fit (violet dashed line). See the main text for explanations.

Next, we add an exponential tail $g\left(i\right)=\gamma e^{-\delta (i-a)}$ starting from a = 59,426. We require f(a)=g(a) and $f^{\prime }\left(a\right)=g^{\prime }\left(a\right)$ in order to have a smooth transition in the neighborhood of *a*. This condition allows us to calculate γ and δ in terms of α and λ, so $g\left(i\right)=f\left(a\right)\cdot e^{-\lambda \frac{i-a}{a}}$. Thus, we obtain augmented avalanche distribution $w_{i}$, consisting of the spline and the exponential tail. All the data and results of the subsequent steps described above are presented in Figure 2. The average size of the avalanche is $<i_{w}>=215$.

**Figure 3:**
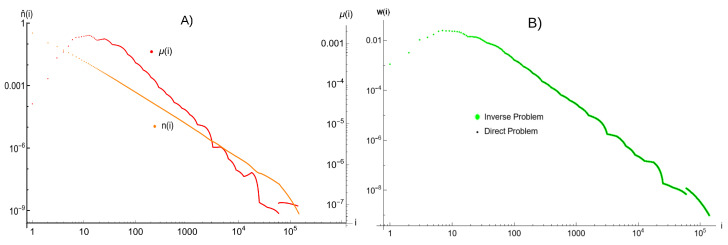
(**A**) Distribution of clusters $n_{i}$ and rebound parameters $\mu_{i}$ obtained from solution of the inverse problem for the augmented distribution of avalanches presented in Figure 2. The left axis refers to the values of $n_{i}$, and the right axis refers to values of the $\mu_{i}$. (**B**) Comparison of the initial distribution of avalanches form Figure 2 with the distribution of avalanches obtained from the solution of the direct problem, i.e., calculated based on the calculated values of $\mu_{i}$. These two distributions overlap in the whole range, which confirms the accuracy of the proposed procedure.

Then, we follow the procedure described in Section 4.2. The value of *I* is set to 1/107, we assume $\hat{c}=1$. The density is ρ≈0.9891589, and ${\hat{x}}_{0}=0.6718844$, ${\hat{x}}_{1}=0.674922$, and ${\hat{x}}_{2}=0.662538$. The obtained distributions of ${\hat{n}}_{i}$ and ${\hat{\mu }}_{i}$, being a solution of the inverse problem, are presented on the left part of Figure 3. It is clear from the picture, that the “jump” of the distribution of avalanches—i.e., a large difference between the values for successive points i = 59,425 and i = 59,426—reflects in the respective “jump” of the distribution of rebound parameters $\mu_{i}$, and the variability of the distribution of clusters remains small.

Next, we calculate the distribution of avalanches $w_{i}$ starting from the obtained distribution of rebounds parameters $\mu_{i}$, i.e., we solve the direct problem. Comparison of the initial and the calculated distributions of avalanches $w_{i}$ is presented in Figure 3 on the right side. These two distributions overlap, confirming the ability of the Random Domino Automaton model to reproduce exact shape of relatively variable distribution of seismic data from LGCD episode.

## 6. Discussion and Conclusions

The article develops the notion of Random Domino Automaton [16] and presents—anticipated earlier in [23]—a solution of the inverse problem for this model. To that aim, we introduced normalized variables (17) and solved the problem algebraically. Then, we applied the procedure to exemplary data [18] recorded in the Legnica-Głogów Copper District in Poland, in order to demonstrate the efficiency of the approach.

The chosen exemplary data are quite challenging, because of the lowered number of records for small magnitudes due to a relatively high catalog completeness threshold and substantial variability for larger magnitudes, due to smaller number of entries. Nevertheless Random Domino Automaton proved to be able to reproduce these variable data very accurately.

We also propose a procedure to deal with a problem of data cut-off. Having recorded data for phenomena following fat-tail distributions, such as, for example, earthquakes, one might expect the appearance of events bigger then those already recorded. From the other side, it is clear that inverse-power distribution can not be valid up to infinity. Thus, we balance these two points by addition of a tail, which smoothly changes from inverse-power to exponential decay.

The next step for extending the RDA model is to introduce aftershocks following the Omori law into the system in order to investigate the waiting time distribution (compare [9]). From the other side, the form of the distribution [8] has been connected with the timing of stress-redistribution events and it leads to so-called anomalous diffusion—see [26,27] and references therein. Extending the RDA model, we aim at providing a mechanistic model connecting the above-mentioned ideas. The solution of the inverse problem for RDA presented in this article is a necessary step for consideration of localization-specific deviations from Gutenberg–Richter law.

Finally, let us note that Random Domino Automaton, in a specific setting, can lead to Motzkin numbers recurrence [28], and give rise to similar construction for more widely known Catalan numbers recurrence [29]. Moreover, in spite of its relatively simple evolution rules, the RDA exhibit complex behavior for which a spontaneous migration between two SOC-like states was discovered [30].

## Data Availability

Data of the Episode LGCD [18] used in Section 5 are open-access seismic data available from IG PAS Data Portal.

## Appendix A. Derivation of the System Equations

Here, we present a complete derivation of the equations of one-dimensional Random Domino Automaton in a general case for arbitrary rebound parameters.

In order to make a description short, we use a symbol $c_{i}\to c_{j}$ to refer to a transition of a cell of one type *i* to another type *j*, and a symbol $\mu_{i}\to c_{0}$ to refer to a transition of an occupied cell to a creating cell, since after an avalanche occupied cells become creating cells only. Occupation of an empty cell of type *i* is denoted by $c_{i}\to \mu_{j}$.

### Appendix A.1. Balance Equation for ρ

In a single time step, the number of occupied cells may stay unchanged (when the new ball is reflected) or may increase by one (when a chosen empty cell becomes occupied) or decrease by *i* (when tan avalanche of size *i* is triggered). Probability rates for specific types of empty cells (i.e., creating, enlarging, merging) to be occupied are

$$\begin{array}{c} c_{0}\to \mu_{i}:\;c_{0}\frac{x_{0}}{N};\;\;c_{1}\to \mu_{i}:\;c_{1}\frac{x_{1}}{N};\;\;c_{2}\to \mu_{i}:\;c_{2}\frac{x_{2}}{N}. \end{array}$$

The probability of relaxation of a cluster of size *i* is $\mu_{i}\left(in_{i}/N\right)$. Clusters of arbitrary size *i* contribute, thus the expected value of the change in the number of occupied cells is

$$\mu_{i}\to c_{0}:\;\sum_{i\geq 1}\frac{n_{i}i}{N}\cdot \mu_{i}\cdot i.$$

Hence, the balance equation for density ρ is

(A1) $$c_{0}x_{0}+c_{1}x_{1}+c_{2}x_{2}=\sum_{i\geq 1}\mu_{i}n_{i}i^{2}.$$

This equation includes no approximations, thus it is exact.

### Appendix A.2. Balance Equation for n

The number of clusters may increase (by 1 in a single time step) only if a new cluster is created, i.e., for $c_{0}\to \mu_{i}$. The number of clusters may decrease (by 1) by the joining of two clusters (i.e., $c_{2}\to \mu_{i}$), and by triggering an avalanche (of arbitrary size), which happens with the probability

$$\sum_{i\geq 1}\mu_{i}\frac{in_{i}}{N}.$$

Hence, the equation is

(A2) $$c_{0}x_{0}-c_{2}x_{2}=\sum_{i\geq 1}\mu_{i}n_{i}i.$$

This equation is exact, too.

### Appendix A.3. The Balance Equations for $x_{i}$’s

In the derivation below it is necessary to take into account the sizes of adjacent clusters and empty clusters, which in our framework are not traced. Thus, from this place, we assume there is *no correlation* between these sizes. This simplification is a version of the *mean field approximation*.

The equation for the number of creating cells $x_{0}$. Four transitions contribute here: avalanche $\mu_{i}\to c_{0}$, a removal of the adjacent cluster $c_{1}\to c_{0}$, occupation of the empty cell, $c_{0}\to \mu_{i}$ and occupation of a neighboring empty cell $c_{0}\to c_{1}$. The expected values (which are the same as probabilities in this case) are

$$\begin{array}{c} c_{1}\to c_{0}:\;x_{1}\sum_{i\geq 1}\frac{n_{i}}{n}\frac{i}{N}\mu_{i},\;\;c_{0}\to c_{1}:\;x_{0}\frac{2}{N}c_{01}, \end{array}$$

where $c_{01}$ is unknown value of probability of occupation of a neighbor of the empty cell of $c_{0}$ type. It is a weighted (somehow) average of $c_{0}$ and $c_{1}$. Note, for $c_{1}\to c_{0}$, a ratio $n_{i}/n$ gives the probability that the neighboring cluster is of size *i*, and $i\mu_{i}/N$ is the probability, which it is removed from the system. Thus, the balance equation for the number of creating cells $x_{0}$ is

(A3) $$\left(c_{0}+2c_{01}\right)x_{0}=\sum_{i\geq 1}\mu_{i}n_{i}i^{2}+\frac{x_{1}}{n}\sum_{i\geq 1}\mu_{i}n_{i}i.$$

The equation for the number of enlarging cells $x_{1}$. The following transitions contribute: $c_{0}\to c_{1}$, $c_{2}\to c_{1}$, $c_{1}\to \mu$, $c_{1}\to c_{0}$, and $c_{1}\to c_{2}$, and because the following rates are

$$\begin{array}{c} c_{2}\to c_{1}:\;2x_{2}\sum_{i\geq 1}\frac{n_{i}}{n}\frac{i}{N}\mu_{i},\;\;c_{1}\to c_{2}:\;x_{1}\frac{1}{N}c_{12}, \end{array}$$

where $c_{12}$ is an unknown value of probability of occupation of a neighbor of the empty cell of $c_{1}$ type. Again, it is a weighted average of $c_{0}$ and $c_{1}$. The equation is

(A4) $$2c_{01}x_{0}-\left(c_{12}+c_{1}\right)x_{1}=\frac{x_{1}-2x_{2}}{n}\sum_{i\geq 1}\mu_{i}n_{i}i.$$

The equation for the number of merging cells $x_{2}$. The following transitions contribute: $c_{1}\to c_{2}$, $c_{2}\to \mu$, and $c_{2}\to c_{1}$, and the equation is

(A5) $$c_{12}x_{1}-c_{2}x_{2}=\frac{2x_{2}}{n}\sum_{i\geq 1}\mu_{i}n_{i}i.$$

**Remark A1.** 
*Not all of the equations derived above are independent. As one may expect, because of the relation (3), a combination of Equations (A3)–(A5) gives the Equation (A1) for the density ρ.*

**Remark A2.** *Because of the relation (7), a combination of Equations (A3)–(A5) must be consistent with the Equation (A2) for the total number of clusters n*.

(A6) $$2c_{01}x_{0}+c_{12}x_{1}=2c_{0}x_{0}+c_{1}x_{1}.$$

For the approximation that there are no correlations between empty cells, both $c_{01}$ and $c_{12}$ are assumed as

(A7) $$c_{01}=c_{12}=\frac{2c_{0}x_{0}+c_{1}x_{1}}{2x_{0}+x_{1}}.$$

It follows from the argument that any given empty cell of $c_{0}$ type is a neighbor for two empty cells (thus contributes twice), while any given empty cell of $c_{1}$ type is a neighbor for only one empty cell. For the Formula (A7), Equation (A6) is satisfied, and a combination of Equations (A4) and (A5) gives Equation (A2) for the total number of clusters *n*.

### Appendix A.4. Equations for the Distribution of Clusters $n_{i}$

The creation of 1-cluster happens with the probability of transition $c_{0}\to \mu_{i}$. The creation of *i*-cluster by enlarging of (i−1)-cluster for i≥2 happens with the probability

$$c_{1}\frac{x_{1}}{N}\frac{n_{i-1}}{n}.$$

The merging of two clusters of size k∈1,2,⋯,(i−2), and of size (i−k−1), for i≥3, separated by a merging cell happens with probability

$$c_{2}\frac{x_{2}}{N}\sum_{k=1}^{i-2}\frac{n_{k}}{n}\frac{n_{i-1-k}}{n}.$$

The removing of *i*-cluster happens with the probability

$$\mu_{i}\frac{in_{i}}{N}.$$

The enlarging of *i*-cluster to any bigger cluster may happen as a result of occupation of neighboring enlarging or merging cell. The respective probabilities are

$$c_{1}\frac{x_{1}}{N}\frac{n_{i}}{n},\;2c_{2}\frac{x_{2}}{N}\frac{n_{i}}{n},$$

where factor 2 comes from the number of neighboring clusters of the empty cell.

Thus, the balance equations for $n_{i}$’s are

(A8) $$\begin{array}{cc} n_{1} & =\frac{1}{\mu_{1}+Y}\left(c_{0}x_{0}\right), \end{array}$$

(A9) $$\begin{array}{cc} n_{2} & =\frac{1}{2\mu_{2}+Y}\left(c_{1}x_{1}\frac{n_{1}}{n}\right), \end{array}$$

(A10) $$\begin{array}{cc} n_{i} & =\frac{1}{i\mu_{i}+Y}\left(c_{1}x_{1}\frac{n_{i-1}}{n}+c_{2}x_{2}\sum_{k=1}^{i-2}\frac{n_{k}}{n}\frac{n_{i-k-1}}{n}\right)\;\mathrm{for}\;i\geq 3, \end{array}$$

where

(A11) $$Y=\frac{1}{n}\left(c_{1}x_{1}+2c_{2}x_{2}\right).$$

**Remark A3.** 
*Equations (A8)–(A10) sum up to balance Equation (A1) for ρ.*

**Remark A4.** 
*It is possible to generalize these equations for the case of a fixed number of possible neighbors bigger then two (RDA on Bethe lattice with coordination number ≥3). For example, for a coordination number equal to 3, the set is supplemented by an analogous equation for i≥4 with an additional triple sum.*
